# Normalized Difference Vegetation Index as a Tool for Wheat Yield Estimation: A Case Study from Faisalabad, Pakistan

**DOI:** 10.1155/2014/725326

**Published:** 2014-06-22

**Authors:** Syeda Refat Sultana, Amjed Ali, Ashfaq Ahmad, Muhammad Mubeen, M. Zia-Ul-Haq, Shakeel Ahmad, Sezai Ercisli, Hawa Z. E. Jaafar

**Affiliations:** ^1^University College of Agriculture, University of Sargodha, Sargodha 40100, Pakistan; ^2^Agro-Climatology Laboratory, Department of Agronomy, University of Agriculture, Faisalabad 38040, Pakistan; ^3^The Patent Office, Karachi 74400, Pakistan; ^4^Department of Agronomy, Bahauddin Zakariya University, Multan 60800, Pakistan; ^5^Department of Horticulture, Ataturk University, 25240 Erzurum, Turkey; ^6^Department of Crop Science, Faculty of Agriculture, University Putra Malaysia, 43400 Selangor, Malaysia

## Abstract

For estimation of grain yield in wheat, Normalized Difference Vegetation Index (NDVI) is considered as a potential screening tool. Field experiments were conducted to scrutinize the response of NDVI to yield behavior of different wheat cultivars and nitrogen fertilization at agronomic research area, University of Agriculture Faisalabad (UAF) during the two years 2008-09 and 2009-10. For recording the value of NDVI, Green seeker (Handheld-505) was used. Split plot design was used as experimental model in, keeping four nitrogen rates (N_1_ = 0 kg ha^−1^, N_2_ = 55 kg ha^−1^, N_3_ = 110 kg ha^−1^, and N_4_ = 220 kg ha^−1^) in main plots and ten wheat cultivars (Bakkhar-2001, Chakwal-50, Chakwal-97, Faisalabad-2008, GA-2002, Inqlab-91, Lasani-2008, Miraj-2008, Sahar-2006, and Shafaq-2006) in subplots with four replications. Impact of nitrogen and difference between cultivars were forecasted through NDVI. The results suggested that nitrogen treatment N_4_ (220 kg ha^−1^) and cultivar Faisalabad-2008 gave maximum NDVI value (0.85) at grain filling stage among all treatments. The correlation among NDVI at booting, grain filling, and maturity stages with grain yield was positive (*R* ^2^ = 0.90; *R* ^2^ = 0.90; *R* ^2^ = 0.95), respectively. So, booting, grain filling, and maturity can be good depictive stages during mid and later growth stages of wheat crop under agroclimatic conditions of Faisalabad and under similar other wheat growing environments in the country.

## 1. Introduction

**Figure 1:**
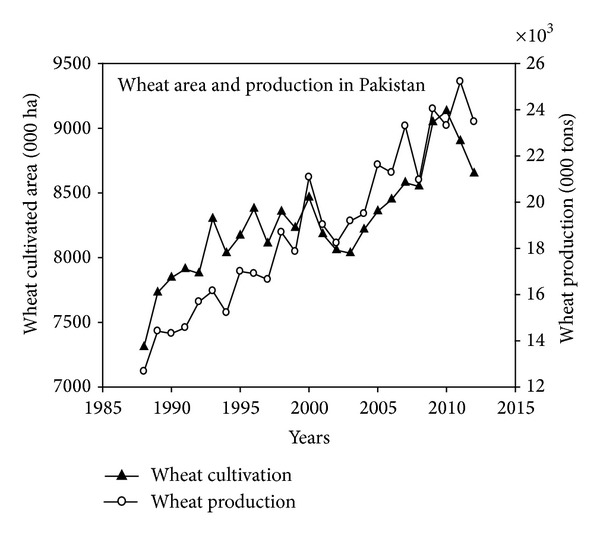
Historical wheat cultivation and production in Pakistan from 1988 to 2012.

Grain crops production plays a dynamic role in the economy of Pakistan with the main crops being wheat, rice, maize, barley, and grams. In 2012, the area under wheat was 8.6 M ha, having production of 24.2 M tons [1]. Wheat contributes 2.2% to gross domestic product (GDP) and 10.1% share in value added agriculture (VAA) in Pakistan. A comparison of historical cultivated area and production is presented in Figure 1 [1]. Given the importance of the wheat crop to the economy of Pakistan, early crop yield forecasting is of vital importance and may help policy makers and grain marketing agencies in planning and management for domestic use and exports.

Normalized Difference Vegetation Index (NDVI) data have been used to monitor crop condition and forecast yield as well as production in many countries of the world, namely, Swaziland [2, 3], Zimbabwe [4], Kenya [5], Spain [6], and Canada [7–9]. Estimation of cereal crops production is a research-based global priority [10] as food grains have a major position in world agricultural production [11]. In semiarid areas, wheat is considerably affected by availability of nitrogen because its deficiency creates nutritional stress, decreases the chlorophyll content in the leaves, and decreases yield [12]. Crop growth research now-a-days is focused on measurement of light interception and its utilization efficiency for evaluation of productivity. As compared to other parameters, near infrared (NIR) based indices are reliable and discerned grain yield meritoriously and results validated the use of spectral reflectance indices (SRI) as an instrument in the breeding programs for the selection increased genetic gain in yield [13].

Application of spectral reflectance and remote sensing technologies for crop growth improvement has encouraged the assessment and appropriate installment of sensors. Vegetation indices, which are determined by the spectral reflectance measurements, are believed to be the most reliable and nondestructive method for effective assessment of total dry matter (TDM) [14, 15], leaf area index (LAI) in wheat and barley [16], and vegetation health and productivity [17]. The estimation of TDM and LAI in the spectral regions of red and near-infrared can be effectively measured by vegetative indices such as NDVI and simple ratio (SR). Normalized Difference Vegetation Index (NDVI) measures the amount of green vegetation in an area. NDVI is based on the principle that actively growing green plants strongly absorb radiation in the visible (VIS) region of the spectrum (the “PAR,” or “photosynthetically active radiation”), while strongly reflecting radiation in the near-infrared (NIR) region. The Green-Seeker Hand Held Optical Sensor Unit is a tool for crop research and consulting and provides precise measurement and data logging of the NDVI and VISr to NIR of plant material. The sensor uses light emitting diodes (LED) to generate red and near-infrared (NIR) light. The light generated is reflected off the crop and measured by a photodiode located at the front of the sensor head. The unit generates light at two specific wavelengths and measures the light reflected off the target (typically plants in soil). The microprocessor within the sensor analyzes the reflected light and calculates the results. The data from the sensor is transmitted serially to an HP iPAQ Personal Digital Assistant and can later be exported to a desktop computer for analysis. Several problems exist when using the sun as a light source. The intensity of sun is affected by sun angle, cloudiness, haziness, and conditions which can cause inconsistent NDVI measurements. NTech developed an active sensor that generates its own illumination source to measure NDVI. In fact, the sensors function in nearly any condition including darkness, dramatically extending the application period. These indices prove to be very helpful in estimation photosynthesizing ability [17] of plants, primary production, and crop yield. No detailed studies exist regarding use of these vegetative indices to differentiate the wheat genotypes according to their response in terms of TDM, LAI, and yield difference in Pakistan. Therefore, this study involving comprehensive range of wheat varieties and their growth assessment at various stages was used to confirm the importance of these vegetative indices on production and yield. The findings will provide an efficient assistance for the management decisions like irrigation or nitrogen application. Application of advanced techniques is a vital step in agronomic experiments for qualitative assessment related to the capability of plants to capture radiation and its utilization in photosynthesis process which can be determined through spectral reflectance [18, 19]. A useful surrogate measurement for the fraction of intercepted radiation throughout a day is the technique of reflectance of light from the crop canopies. Evaluation of plant initial biomass and strength in various wheat varieties can be efficiently secured through RSI [16, 20]. Therefore, awareness should be created for utilization of modern techniques like spectral reflectance or remote sensing to help agronomist, farm managers, and farmers to cope with abiotic stresses timely. The objectives of this study, therefore, were (a) to study effect of various nitrogen rates and wheat cultivars on NDVI in Faisalabad district and (b) to identify the best growth stages for making a reliable crop yield forecast.

## 2. Materials and Methods

The experiments were laid out at Agronomic Research Area, University of Agriculture Faisalabadin with randomized complete block design (RCBD) with split plot arrangement having four replications. The plot size was 10 m × 2.4 m. The experiments considered ten cultivars (Bakkhar-2001, Chakwal-50, Chakwal-97, Faisalabad-2008, GA-2002, Inqlab-91, Lasani-2008, Miraj-2008, Sahar-2006, Shafaq-2006) in subplots and four nitrogen levels (0, 55, 110, and 220 kg ha^−1^) in main plots. The wheat crop was sown on 12th November during both the years of 2008-2009 and 2009-2010 with the help of single row hand drill, keeping row to row distance of 30 cm. The phosphorus and potassium were applied at the rate of 85 and 60 kg ha^−1^, respectively. Urea, triple supper phosphate, and sulphate of potash were used as sources of N, P, and K fertilizers, respectively. The potash and phosphorus fertilizers were applied at the time of sowing, while the N was top dressed in two splits. Cultural practices such as weeding and irrigation were kept uniform for all the experimental treatments. Two equal splits of nitrogen fertilizer were applied first at 35 (17th December) and 60 (11th January) days after sowing (DAS), respectively, during both the years [21]. A total of 19 acre inches of water were applied; four acre inches for seed bed preparation, three acre inches each at tillering, stem elongation, booting, anthesis, and grain formation stages [21].

### 2.1. Soil Analysis and Weather Data

Composite soil samples were taken at the experiment site prior to seeding. The samples were analyzed for major physical and chemical soil properties by standard methods (Table 1). The soil is sandy clay loam according to USDA classification. Its color is brown, somewhat poorly drained. The mean monthly weather data for both years are presented in Table 2.

### 2.2. Observations Recorded

Spectral reflectance was measured by a spectroradiometer (Green-Seeker Hand Held optical sensor unit, model 505; NTech Industries, Inc., Ukiah, CA, USA), above the canopy at 50 cm height at different growth stages during the season. Each plot was divided into two subplots. One of them was used for destructive biomass and leaf area sampling and the other remained intact for reflectance measurements and final grain yield determination. Total dry matter (TDM) and NDVI were measured on fortnightly basis at tillering, stem elongation, booting, anthesis, grain filling, and maturity. Half meter long row from each plot was harvested at ground level after every twenty days interval leaving appropriate borders. Then 5 g of green leaf laminae was used to record leaf area on leaf area meter (Model CI-202, CID, Inc.). Fresh and dry weight of component fraction of plant (leaf and stem) was determined. A subsample in each fraction was taken to dry in an oven at 70°C to a constant weight.

### 2.3. Calculation of Vegetation Indices

Initially, different ratios and normalized indices were determined based on a combination of visible and near-infrared wavelength, as suggested by scientists [13]. NDVI was obtained with the following expression:

(1) $$\begin{array}{c} \text{NDVI}=\frac{\left(\text{NIR}-\text{VISr}\right)}{\left(\text{NIR}+\text{VISr}\right)}, \end{array}$$

where NDVI stands for normalized difference vegetation index, NIR for near-infrared radiation, and VISr for visible red spectrum. NDVI values range from −1 (usually water) to +1 (strongest vegetative growth). The amount of reflectance in the NIR range (*λ* = 700–1300 nm) and in the VISr range (*λ* = 550–700 nm) is determined by the optical properties of the leaf tissues: their cellular structure and the air-cell wall-protoplasm-chloroplast interfaces [22]. A portable spectroradiometer known as Green-Seeker (Hand Held Optical Sensor Unit, Model 505; NTech Industries, INC., Ukiah, CA, USA) was used to measure NDVI.

## 3. Results and Discussion

### 3.1. NDVI at Various Growth Stages

Results showed that effect of nitrogen on NDVI score at various growth stages, that is, tillering, stem elongation, booting, anthesis, grain filling and physiological maturity was significant (Table 4). Maximum values of NDVI were observed in N_4_ (220 kg N ha^−1^) and minimum values were showed in N_1_ (0 kg N ha^−1^) at different growth stages in chronological order, respectively, (Table 4). Other treatments of nitrogen N_3_ (110 kg N ha^−1^) and N_2_ (55 kg N ha^−1^) showed values of NDVI score between above mentioned range. Similarly, variation among cultivars differed significantly at all growth stages. In chronological order, NDVI score ranged from 0.32 to 0.43, 0.53 to 0.70, 0.55 to 0.74, 0.74 to 0.85, 0.78 to 0.88, and 0.40 to 0.65 at tillering, stem elongation, booting, anthesis, grain filling and physiological maturity stages, respectively. The interaction of between nitrogen rates and cultivars for NDVI was highly significant at maturity stage and data presented in Tables 5 and 6 for years 2008-09 and 2009-10, respectively, while, non-significant at all other growth stages (Table 4). Many workers [23, 24] reported that at grain filling stage NDVI decreases up to 0.3 because crop becomes under stressed and its capacity to absorb PAR is reduced. But others [25, 26] described that NDVI score reached up to 0.4 in productive environments which have high TDM and LAI thus showing the vigorous crop canopy as dark foliage. The milky-grain stage is the best depictive stage for recording NDVI as it directly correlates to yield than earlier measurements [27]. More of the literature showed that the relationship between biomass and NDVI but less information for prediction of biomass and yield at early growth stages of the crop consistent with the findings of other group of scientists [13, 28], NDVI score increased until the onset of grain filling and the highest score of NDVI recorded near milky-grain stage and then decrease up to maturity. Biomass and yield was strongly correlated with each other.

### 3.2. Time-Course Vegetation Indices and Peak LAI

Changes in LAI throughout the growth cycle were reflected in the vegetation index. Thus, NDVI reached a maximum from 106 to 126 DAS and started to decline from this date onwards (Table 4). However, in all the treatments including 220 kg N ha^−1^, NDVI declined abruptly at maturity (Table 4). The peak LAI was the highest at 220 kg N ha^−1^ and lowest value was recorded at control (0 kg N ha^−1^), and peak LAI was measured at booting stage (Table 3). Many researchers [15, 25, 29, 30] concluded that increases in red reflectance were related to the decreases in chlorophyll content resulting from lower N supply [31], decreases in NIR reflectance mostly responded to decreases in LAI and green biomass, as has been widely reported for wheat crops.

### 3.3. Correlation Coefficient between Grain Yield and NDVI Score at Different Growth Stages

Table 7 shows the simple correlation between grain yield and NDVI scores. At the time of tillering, stem elongation, booting, anthesis, grain filling and maturity, these relationships were highly positively correlated in both seasons, and significant association between grain yield and NDVI was also found having maximum value at maturity stage.

### 3.4. NDVI Trend during the Crop Growth Period

**Figure 2:**
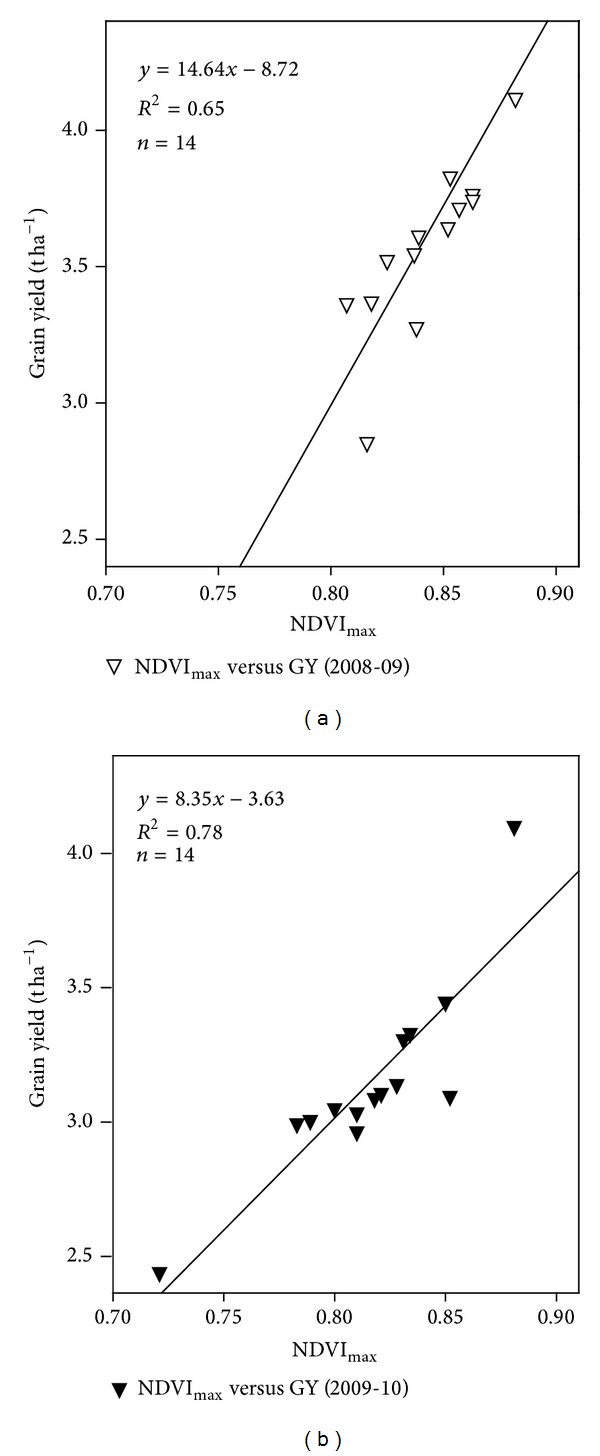
Relationship between grain yield and NDVI_max⁡_.

**Figure 3:**
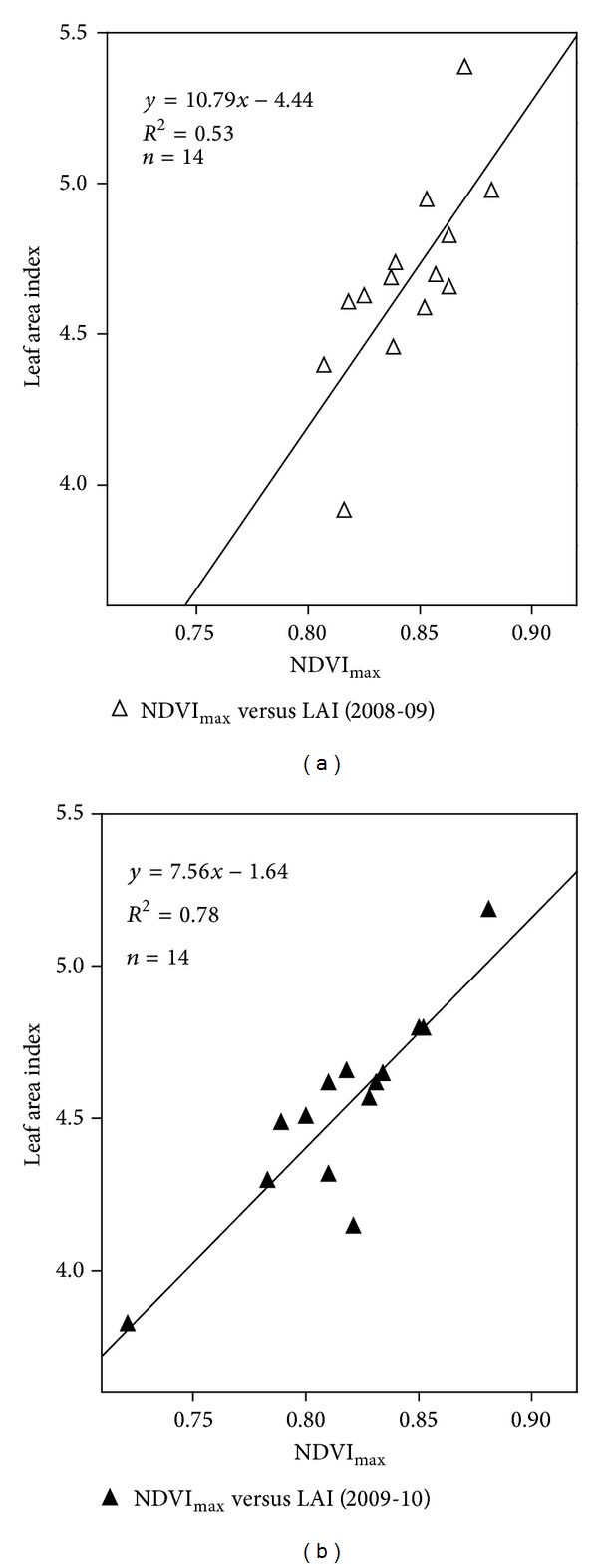
Relationship between leaf area index and NDVI_max⁡_.

Wheat grown under different N levels, showed only small differences in NDVI among cultivars once 100% ground cover was reached, but variability increased as the crop cycle progressed and highest variability was obtained at maximum head weight due to differences in spike size and/or morphology. The difference among cultivars increased for the prediction of grain yield at heading and grain filling stages, quite likely due to a decrease in LAI and differing morphological characteristics of spike. There was a positive and linear relationship between grain yield and maximum NDVI (Figure 2). Regression accounted for 65% and 78% variations of the data during both the years of study. Relationship between LAI and maximum NDVI is positive and linear (*R* ^2^ = 0.53 and 0.78) during years 2008-09 and 2009-10, respectively, as Figure 3 shows.

## 4. Conclusion

The potential of NDVI to differentiate wheat cultivars for grain yield under different nitrogen levels was demonstrated. The NDVI was able to differentiate cultivars at different growth stages. NDVI scores at the booting, grain filling, anthesis, and maturity stages can be used as yield predictors in wheat and overall; the study showed a clear association between grain yield and NDVI measured but at maturity stage correlation between grain yield and NDVI was greater than NDVI values recorded at different growth stages.

## Figures and Tables

**Table 1:** Physical and chemical analysis of soil.

Chemical properties	2008-09	2009-10
Organic matter	1.28%	1.23%
TTS (total soluble salt)	12.29%	11.69
pH	7.54	7.21
Nitrogen (N)	0.64%	0.57
Phosphorus (P_2_O_5_)	6.93 ppm	6.85 ppm
Potassium (K)	19.4 ppm	19.1 ppm

**Table 2:** Weather data at experimental site.

Months	Rainfall (mm)		Temperature (°C) 2008-09		Temperature (°C) 2009-10		Sunshine (hours)	
	2008	2009	max.	min.	max.	min.	2008	2009
November	0	0.7	27.3	12.2	25.7	10.8	8.0	06.3
December	14.6	0	21.9	09.1	22.1	07.0	6.0	06.6
January	13.5	0.8	19.6	07.3	16.2	06.0	6.1	04.1
February	18.2	11.9	22.1	09.9	22.0	09.5	7.3	06.6
March	14.0	8.8	27.5	14.0	30.4	16.5	7.8	08.7
April	22.9	1.3	33.5	19.1	38.4	21.4	9.2	09.0

**Table 3:** Effect of nitrogen levels and cultivars on peak LAI at booting stage.

Treatments	LAI	
	2008-09	2009-10
Nitrogen levels		
0 kg ha^−1^	3.91^d^	3.82^d^
55 kg ha^−1^	4.45^c^	4.31^c^
110 kg ha^−1^	4.94^b^	4.79^b^
220 kg ha^−1^	5.38^a^	5.18^a^
LSD (0.05)	0.32	0.25
Cultivars		
Faisalabad-2008	4.97^a^	4.79^a^
Lasani-2008	4.82^ab^	4.64^ab^
Miraj-2008	4.69^b^	4.56^ab^
Sahar-2006	4.65^bc^	4.61^ab^
Shafaq-2006	4.68^b^	4.61^ab^
GA-2002	4.39^c^	4.29^cd^
Bakkhar-2001	4.60^bc^	4.48^bc^
Inqlab-1991	4.73^ab^	4.65^ab^
Chakwal-1950	4.58^bc^	4.14^d^
Chakwal-1997	4.62^bc^	4.50^bc^
LSD (0.05)	0.25	0.25

Means sharing different letters differ significantly at *P* ≤ 0.05.

**Table 4:** Effect of varying nitrogen levels and wheat cultivars on NDVI score at different growth stages.

Treatment	Tillering		Stem elongation		Booting		Anthesis		Grain filling		Maturity	
	2008-09	2009-10	2008-09	2009-10	2008-09	2009-10	2008-09	2009-10	2008-09	2009-10	2008-09	2009-10
Nitrogen levels (N)												
0 kg ha^−1^	0.31^c^	0.34^d^	0.53^c^	0.51^b^	0.53^d^	0.51^c^	0.73^c^	0.64^c^	0.82^c^	0.72^b^	0.49^c^	0.34^d^
55 kg ha^−1^	0.37^b^	0.37^c^	0.58^bc^	0.64^a^	0.58^c^	0.62^b^	0.78^b^	0.76^b^	0.84^bc^	0.81^a^	0.52^c^	0.40^c^
110 kg ha^−1^	0.41^ab^	0.41^b^	0.63^ab^	0.64^a^	0.62^b^	0.72^a^	0.80^ab^	0.83^ab^	0.85^ab^	0.85^a^	0.59^b^	0.49^b^
220 kg ha^−1^	0.44^a^	0.47^a^	0.65^a^	0.66^a^	0.73^a^	0.73^a^	0.82^a^	0.87^a^	0.87^a^	0.88^a^	0.65^a^	0.61^a^
DMR (5%)	0.05	0.03	0.06	0.06	0.03	0.05	0.03	0.10	0.03	0.09	0.03	0.06
Significance	∗∗	∗∗	∗∗	∗∗	∗∗	∗∗	∗∗	∗∗	∗∗	∗∗	∗∗	∗∗
S$\overset{-}{x}$	0.012	0.006	0.013	0.013	0.006	0.010	0.006	0.022	0.006	0.019	0.013	0.014
Varieties (V)												
Faisalabad-2008	0.42^a^	0.43^a^	0.67^a–c^	0.70^a^	0.69^a^	0.74^a^	0.81^a^	0.85^a^	0.88^a^	0.85^a^	0.65^a^	0.53^a^
Lasani-2008	0.41^a^	0.42^ab^	0.64^bc^	0.68^ab^	0.66^ab^	0.70^ab^	0.81^ab^	0.82^ab^	0.86^ab^	0.83^ab^	0.61^ab^	0.49^ab^
Miraj-2008	0.39^ab^	0.40^a–c^	0.60^a–c^	0.62^a–c^	0.61^cd^	0.66^bc^	0.79^a–c^	0.77^cd^	0.86^ab^	0.83^ab^	0.58^bc^	0.48^b–d^
Sahar-2006	0.39^ab^	0.41^a–c^	0.61^bc^	0.62^a–c^	0.62^bc^	0.71^ab^	0.80^a–c^	0.79^bc^	0.86^e^	0.83^ab^	0.59^ab^	0.48^a–c^
Shafaq-2006	0.38^a–c^	0.39^a–c^	0.58^ab^	0.60^a–c^	0.59^cd^	0.64^c^	0.78^bc^	0.74^d^	0.84^a–c^	0.81^a–c^	0.54^b–d^	0.44^b–e^
GA-2002	0.33^c^	0.35^d^	0.54^d^	0.53^c^	0.57^d^	0.55^e^	0.74^d^	0.74^d^	0.81^b–e^	0.78^c^	0.49^d^	0.40^e^
Bakkhar-2001	0.35^c^	0.38^cd^	0.57^a–c^	0.57^bc^	0.59^cd^	0.56^de^	0.77^c^	0.74^d^	0.82^de^	0.79^bc^	0.51^cd^	0.42^de^
Inqlab-91	0.38^a–c^	0.39^a–c^	0.58^c^	0.60^a–c^	0.60^cd^	0.62^c^	0.78^a–c^	0.75^d^	0.84^b–d^	0.82^a–c^	0.56^b–d^	0.47^b–d^
Chakwal-50	0.39^ab^	0.40^a–c^	0.60^bc^	0.61^a–c^	0.58^cd^	0.64^c^	0.79^a–c^	0.77^cd^	0.85^c–e^	0.82^a–c^	0.57^bc^	0.47^b–d^
Chakwal-97	0.36^a–c^	0.38^b–d^	0.57^bc^	0.59^bc^	0.57^cd^	0.61^cd^	0.77^c^	0.75^d^	0.83^ab^	0.80^bc^	0.53^b–d^	0.43^c–e^
DMR (5%)	0.05	0.04	0.07	0.10	0.04	0.05	0.03	0.04	0.03	0.04	0.07	0.05
Significance	∗∗	∗∗	NS	∗	∗∗	∗∗	∗∗	∗∗	∗∗	∗∗	∗∗	∗∗
S$\overset{-}{x}$	0.016	0.013	0.022	0.032	0.012	0.016	0.009	0.013	0.009	0.013	0.020	0.016
Interaction (N × V)	NS	NS	NS	NS	∗∗	NS	NS	NS	NS	NS	∗∗	∗∗
Mean	**0.38**	**0.40**	**0.59**	**0.61**	**0.61**	**0.66**	**0.78**	**0.77**	**0.84**	**0.82**	**0.5625**	**0.459**

Means not sharing any two letters differ significantly at (*P* ≤ 0.05); ∗significant at 5% level; ∗∗significant at 1% level; NS: nonsignificant.

**Table 5:** Interactive effect of nitrogen levels in wheat cultivars for NDVI score at maturity stage for the year 2008-09.

Wheat cultivars	Nitrogen levels				Means
	N_1_ (0 kg ha^−1^)	N_2_ (55 kg ha^−1^)	N_3_ (110 kg ha^−1^)	N_4_ (220 kg ha^−1^)	
V_1_ = Faisalabad-2008	0.56^a^	0.59^a^	0.68^a^	0.74^a^	0.65^A^
V_2_ = Lasani-2008	0.56^a^	0.48^ab^	0.67^a^	0.71^a^	0.61^AB^
V_3_ = Miraj-2008	0.51^ab^	0.57^ab^	0.58^ab^	0.68^a^	0.58^BC^
V_4_ = Sahar-2006	0.53^ab^	0.49^ab^	0.65^ab^	0.67^a^	0.59^AB^
V_5_ = Shafaq-2006	0.55^a^	0.48^ab^	0.50^b^	0.63^ab^	0.54^B–D^
V_6_ = GA-2002	0.37^bc^	0.43^b^	0.53^b^	0.64^ab^	0.49^D^
V_7_ = Bakkhar-2001	0.26^c^	0.58^a^	0.67^a^	0.55^b^	0.51^CD^
V_8_ = Inqlab-91	0.57^a^	0.58^a^	0.59^ab^	0.49^c^	0.56^B–D^
V_9_ = Chakwal-50	0.41^b^	0.49^ab^	0.67^a^	0.71^a^	0.57^BC^
V_10_ = Chakwal-97	0.58^a^	0.53^ab^	0.33^c^	0.70^a^	0.53^B–D^
Means	**0.49** ^ C^	**0.52** ^ C^	**0.59** ^ B^	**0.65** ^ A^	

Means sharing different letters differ significantly at *P* ≤ 0.05.

**Table 6:** Interactive effect of nitrogen levels wheat cultivars for NDVI score at maturity stage for the year 2009-10.

Wheat cultivars	Nitrogen levels				Means
	N_1_ (0 kg ha^−1^)	N_2_ (55 kg ha^−1^)	N_3_ (110 kg ha^−1^)	N_4_ (220 kg ha^−1^)	
V_1_ = Faisalabad- 2008	0.37^ab^	0.44^a^	0.57^a^	0.74^a^	0.53^A^
V_2_ = Lasani-2008	0.35^ab^	0.44^a^	0.55^ab^	0.60^bc^	0.49^AB^
V_3_ = Miraj-2008	0.35^ab^	0.44^a^	0.47^b^	0.64^b^	0.48^B–D^
V_4_ = Sahar-2006	0.37^a^	0.45^a^	0.52^ab^	0.59^bc^	0.48^A–C^
V_5_ = Shafaq-2006	0.28^b^	0.40^ab^	0.45^b^	0.62^b^	0.44^B–E^
V_6_ = GA-2002	0.35^ab^	0.31^b^	0.41^b^	0.52^c^	0.40^E^
V_7_ = Bakkhar-2001	0.37^a^	0.39^ab^	0.43^b^	0.51^c^	0.42^DE^
V_8_ = Inqlab-1991	0.33^ab^	0.43^ab^	0.52^ab^	0.60^bc^	0.47^B–D^
V_9_ = Chakwal-1950	0.33^ab^	0.34^b^	0.48^b^	0.73^a^	0.47^B–D^
V_10_ = Chakwal-1997	0.26^b^	0.39^ab^	0.50^ab^	0.56^bc^	0.43^C–E^
Means	**0.34** ^ D^	**0.40** ^ C^	**0.49** ^ B^	**0.61** ^ A^	

Means sharing different letters differ significantly at *P* ≤ 0.05.

**Table 7:** Correlation coefficient between grain yield and NDVI score at different growth stages.

Development stages	2008-2009 (*n* = 14)	2009-2010 (*n* = 14)	Pooled (*n* = 28)
Tillering	0.928∗∗	0.925∗∗	0.936∗∗
Stem elongation	0.880∗∗	0.694∗	0.796∗∗
Booting	0.950∗∗	0.707∗∗	0.901∗∗
Anthesis	0.866∗∗	0.879∗∗	0.909∗∗
Grain filing	0.805∗∗	0.881∗∗	0.901∗∗
Maturity	0.927∗∗	0.950∗∗	0.959∗∗

*Significant at 5% level. **Significant at 1% level.

## References

[1] Government of Pakistan (2013). *Economic Survey of Pakistan, 2012-2013*.

[2] Mkhabela MS, Mkhabela MS (2000). Exploring the possibilities of using NOAAAVHRR data to forecast cotton yield in Swaziland. *UNISWA Journal of Agriculture*.

[3] Mkhabela MS, Mkhabela MS, Mashinini NN (2005). Early maize yield forecasting in the four agro-ecological regions of Swaziland using NDVI data derived from NOAA’s-AVHRR. *Agricultural and Forest Meteorology*.

[4] Unganai LS, Kogan FN (1998). Drought monitoring and corn yield estimation in Southern Africa from AVHRR data. *Remote Sensing of Environment*.

[5] Lewis JE, Rowland J, Nadeau A (1998). Estimating maize production in Kenya using NDVI: some statistical considerations. *International Journal of Remote Sensing*.

[6] Vicente-Serrano S, Cuadrat-Prats JM, Romo A (2006). Early prediction of crop production using drought indices at different time-scales and remote sensing data: application in the Ebro Valley (North-East Spain). *International Journal of Remote Sensing*.

[7] Bullock PR (1992). Operational estimates of Western Canadian grain production using NOAA AVHRR LAC data. *Canadian Journal of Remote Sensing*.

[8] Boken VK, Shaykewich CF (2002). Improving an operational wheat yield model using phenological phase-based Normalized Difference Vegetation Index. *International Journal of Remote Sensing*.

[9] Wall L, Larocque D, Pierre-Majorique L (2008). The early explanatory power of NDVI in crop yield modelling. *International Journal of Remote Sensing*.

[10] Steinmetz S, Guerif M, Delecolle R, Baret F (1990). Spectral indices. *Remote Sensing of Environment*.

[11] Rudorff BFT, Mulchi CL, Daughtry CST, Lee EH (1996). Growth, radiation use efficiency, and canopy reflectance of wheat and corn grown under elevated ozone and carbon dioxide atmospheres. *Remote Sensing of Environment*.

[12] Karimpour M, Siosemardeh A, Fateh H, Badakhshan H, Heidari G (2013). Effects of nitrogen fertilizer on yoeld and som physiological charachteristics on two drought resistance and susceptible wheat (*Tritticum aestivum* L.) cultivars in response to water stress. *International Journal of Farming and Allied Sciences*.

[13] Babar MA, Reynolds MP, van Ginkel M, Klatt AR, Raun WR, Stone ML (2006). Spectral reflectance to estimate genetic variation for in-season biomass, leaf chlorophyll, and canopy temperature in wheat. *Crop Science*.

[14] Tucker CJ (1979). Red and photographic infrared linear combinations for monitoring vegetation. *Remote Sensing of Environment*.

[15] Asrar G, Fuchs M, Kanemasu ET, Morgan JL (1984). Estimating absorbed photosynthetic radiation and leaf area index from spectral reflectance in wheat. *Agronomy Journal*.

[16] Elliott GA, Regan KL (1993). Use of reflectance measurements to estimate early cereal biomass production on sand plain soils. *Australian Journal of Experimental Agriculture*.

[17] Wang J, Rich PM, Price KD, Kettle WD (2005). Relations between NDVI, grassland production, and crop yield in the central great plains. *Geocarto International*.

[18] Ma BL, Morrison MJ, Dwyer LM (1996). Canopy light reflectance and field greenness to assess nitrogen fertilization and yield of maize. *Agronomy Journal*.

[19] Teal RK, Tubana B, Girma K (2006). In-season prediction of corn grain yield potential using normalized difference vegetation index. *Agronomy Journal*.

[20] Bellairs M, Turner NC, Hick PT, Smith CG (1996). Plant and soil influences on estimating biomass of wheat in plant breeding plots using field spectral radiometers. *Australian Journal of Agriculture Research*.

[21] Sultana SR, Ahmad A, Wajid A, Akhtar J (2013). Estimating growth and yield related traits of wheat genotypes under variable nitrogen application in semi-arid conditions. *Pakistan Journal of Life and Social Sciences*.

[22] Kumar R, Silva L (1973). Light ray tracing through a leaf section. *Applied Optics*.

[23] Gamon JA, Peňuelas J, Field CB (1992). A narrow-waveband spectral index that tracks diurnal changes in photosynthetic efficiency. *Remote Sensing of Environment*.

[24] Gamon JA, Serrano L, Surfus J (1997). The photochemical reflectance index: an optical indicator of photosynthetic radiation use efficiency across species, functional types, and nutrient levels. *Oecologia*.

[25] Fernández S, Vidal D, Simón E, Solé-Sugraňes L (1994). Radiometric characteristics of *Triticum aestivum* cv.Astral under water and nitrogen stress. *International Journal of Remote Sensing*.

[26] Wanjura DF, Hatfield JL (1987). Sensitivity of spectral vegetative indices to crop biomass. *Transactions of the American Society of Agricultural Engineers*.

[27] Royo C, Aparicio N, Villegas D, Casadesus J, Monneveux P, Araus JL (2003). Usefulness of spectral reflectance indices as durum wheat yield predictors under contrasting Mediterranean conditions. *International Journal of Remote Sensing*.

[28] Babar MA, Reynolds MP, van Ginkel M, Klatt AR, Raun WR, Stone ML (2006). Spectral reflectance indices as a potential indirect selection criteria for wheat yield under irrigation. *Crop Science*.

[29] Jensen A, Lorenzen B, Spelling-Ostergaard H, Kloster- Hvelplund E (1990). Radiometric estimation of biomass and nitrogen content of barley grown at different nitrogen levels. *International Journal of Remote Sensing*.

[30] Penuelas J, Filella I, Serrano L, Save R (1996). Cell wall elasticity and Water Index (R970 nm/R900 nm) in wheat under different nitrogen availabilities. *International Journal of Remote Sensing*.

[31] Filella I, Serrano L, Serra J, Penuelas J (1995). Evaluating wheat nitrogen status with canopy reflectance indices and discriminant analysis. *Crop Science*.

